# From Black Box to Transparency: The Impact of Multi-Level Visualization on User Trust in Autonomous Driving

**DOI:** 10.3390/s25216725

**Published:** 2025-11-03

**Authors:** Mengniu Li, Ming Zhou, Yajun Li, Wentao Wei, Tianlu Zhu, Xun Xu, Linyan Ren, Nuowen Zhang, Renhan Xu, Jinye Li

**Affiliations:** 1 School of Design Art & Media, Nanjing University of Science and Technology, Nanjing 210094, China; limengniu@njust.edu.cn (M.L.); lyj5088@163.com (Y.L.); weiwentao@njust.edu.cn (W.W.); 220109011180@njust.edu.cn (T.Z.); xuxun821@ecut.edu.cn (X.X.); renly@njust.edu.cn (L.R.); katherinaz@126.com (N.Z.); 2 Nanjing Institute of Electronic Equipment, Nanjing 210007, China; xrh10.00@126.com; 3 College of Life and Ocean Science, Shenzhen University, Shenzhen 518057, China; 2450171015@mails.szu.edu.cn

**Keywords:** autonomous driving, human–machine trust, meaningful transparency, explainable visualization, cognitive load, eye-tracking

## Abstract

**Highlights:**

This study employs a simulated driving experiment to systematically compare the effects of three visualization conditions—black-box, standard, and enhanced—on drivers’ trust, cognitive load, and behavioral responses, integrating multimodal data from eye-tracking, manual interventions, and questionnaire assessments.

**What are the main findings?**

- Enhanced visualization significantly improves users’ functional trust and perceived usefulness in autonomous driving systems.
- The black-box system does not reduce physiological stress, but instead leads to an “out-of-the-loop” state, characterized by cognitive withdrawal and monitoring complacency.

**What is the implication of the main finding?**

- Demonstrates that “meaningful transparency” is key to establishing appropriate human–machine trust, outperforming designs that are either completely opaque or involve information overload.
- Provides a trust assessment basis and optimization direction for autonomous driving human–machine interface design, grounded in eye-tracking and behavioral data.

**Abstract:**

Autonomous systems’ “black-box” nature impedes user trust and adoption. To investigate explainable visualizations’ impact on trust and cognitive states, we conducted a within-subjects study with 29 participants performing high-fidelity driving tasks across three transparency conditions: black-box, standard, and enhanced visualization. Multimodal data analysis revealed that enhanced visualization significantly increased perceived usefulness by 28.5% (*p* < 0.001), improved functional trust, and decreased average pupil diameter by 15.3% (*p* < 0.05), indicating lower cognitive load. The black-box condition elicited minimal visual exploration, lowest subjective ratings, and “out-of-the-loop” behaviors. Fixation duration showed no significant difference between standard and enhanced conditions. These findings demonstrate that well-designed visualizations enable balanced trust calibration and cognitive efficiency, advocating “meaningful transparency” as a core design principle for effective human–machine collaboration in autonomous vehicle interfaces. This study provides empirical evidence that transparency enhances user experience and system performance.

## 1. Introduction

Advances in electronic and information technologies have accelerated the development of automotive automation. The Society of Automotive Engineers (SAE) International defines a maturity scale for autonomous driving (Levels L0–L5), spanning from fully manual to fully automated operation [1]. Currently, the majority of passenger vehicles worldwide operate at Level 2 (partial automation, requiring continuous driver supervision), while select high-end models have achieved Level 3 (conditional automation, wherein the system assumes control under predefined conditions). Level 4 systems (high automation) are undergoing commercial pilot deployments in select regions—such as Waymo’s operations in Phoenix and Baidu Apollo’s trials in Chinese cities—while Level 5 (full automation) remains confined to laboratory environments [2,3,4,5,6].

In practice, end-to-end deep learning models—while efficient at processing complex environments—are inherently black-box, rendering their decision-making logic difficult to trace. However, eXplainable Artificial Intelligence (XAI) tools enable systems to generate local explanations for drivers or regulators, thereby enhancing comprehension of critical decisions [7,8,9].

The complexity of autonomous driving systems stems from their heavy reliance on fusing multi-source sensor data. Early, mid-, and late-stage fusion strategies collectively support environmental perception, path planning, and decision execution [10,11]. Nevertheless, this complexity renders system decision logic “opaque” to users—even when supplemented with local explanations—triggering a trust crisis [12]. Consequently, the trust gap between autonomous systems and human operators has emerged as a core bottleneck to large-scale commercialization, exacerbated by opaque and redundant information displays in human–machine interfaces that further elevate cognitive load and perceived uncertainty [13]. Establishing and calibrating appropriate user trust in black-box systems now constitutes a pivotal technical challenge and a central concern in human factors engineering for autonomous driving.

### 1.1. Background

Some scholars argue that users can establish trust through social consensus and safety assurances without needing to understand the inner workings of black-box systems [14]. Heitor’s study found that users exhibit high trust in black-box systems, and explainability elements do not significantly influence trust behaviors [15]. Domain experts place greater value on high-quality explanations, whereas lay users exhibit lower demand for such detail. Concise, task-relevant explanations tend to enhance trust more effectively, while overly complex or inconsistent explanations may erode it [16,17]. In certain domains, XAI does not significantly improve participants’ self-reported trust in AI systems [18]. Trust cultivated through communication, education, and training proves more robust and enduring than trust derived solely from system explainability [19].

In contrast, other studies emphasize that users prioritize safety and controllability above all else [20,21]. Trust grounded solely in social consensus or system performance often falls short of users’ expectations for reliability. Transparency and explainability are still considered essential prerequisites for earning user trust in AI systems—particularly in safety-critical domains [22,23,24]. Enhancing the transparency and explainability of autonomous driving systems thus remains a key challenge in trust building. Researchers commonly employ semantic text explanations: Dong et al. [25] frame driving decisions as image captioning tasks, using Transformer models to generate meaningful sentences describing driving scenes, thereby explaining the rationale behind driving behaviors. Zhang et al. [26] leverage attention-based modules to capture interactions between the ego vehicle and surrounding traffic objects, generating semantic textual explanations.

Beyond textual approaches, visualization techniques such as augmented reality (AR) and high-fidelity 3D rendering also enhance users’ understanding of system states and decision logic, effectively reducing anxiety toward “black-box” systems and improving user experience and trust [27,28,29,30]. For instance, automakers like Tesla and academic research teams have employed Unreal Engine to optimize visualization systems, enhancing environmental realism and enabling users to intuitively perceive how the system interprets its surroundings [31,32]. Tobias et al. [33] overlaid color-coded blocks and lane indicators onto driving simulators via AR, providing explanations during or after driving; results showed such interventions could transform negative experiences into neutral ones. High-fidelity, interactive visual feedback not only improves situational awareness and perceived safety, but also enables better trust calibration by visually conveying system uncertainty and decision rationale in real time [34]. However, information presentation must balance richness with cognitive load, avoiding user confusion or over-trust due to information overload [35].

Current research widely employs multimodal physiological signals, behavioral metrics, and self-report questionnaires to assess users’ cognitive states. Lu et al. [36] successfully used eye-tracking to infer trust levels in real time. Wang et al. [37] collected drivers’ eye movement data via the ETG-2w portable eye-tracking glasses and measured takeover reaction time by recording when drivers pressed the “takeover” button. J. Choi and Y. Ji [38] constructed a survey based on an extended Technology Acceptance Model (TAM) to explore how perceived usefulness, ease of use, trust, perceived risk, and system transparency influence behavioral intention to use autonomous driving systems.

### 1.2. Research Motivation

In the context of trust-building for high-risk AI systems such as autonomous driving, the academic community remains divided between two seemingly opposing perspectives—reflecting a fundamental debate over whether users need to understand system behavior to trust it. Automotive manufacturers face a strategic dilemma: should R&D resources be prioritized toward improving the underlying system safety, or allocated to developing costly, complex visualization-based explanation systems [39]? Is explainable visualization a functional necessity for user trust, or merely a value-added feature for marketing appeal? Moreover, when users self-report increased trust, does this subjective perception genuinely align with their cognitive states and behavioral responses?

Much of the existing literature relies heavily on self-report scales, which are vulnerable to cognitive biases and social desirability effects. For instance, users may verbally express confidence in a system while instinctively intervening during critical moments—a behavioral contradiction that reveals a potential disconnect between stated trust and actual reliance. Objective measures—such as physiological responses and behavioral interventions—may offer more reliable indicators of users’ true cognitive and affective states when interacting with “black-box” systems. These metrics are critical for empirically evaluating the competing paradigms of “black-box acceptability” versus “transparency necessity.” For example: Under a no-visualization condition (Level 0), do users exhibit higher intervention rates or physiological arousal in response to system decisions? Conversely, does enhanced visualization (Level 2) lead to measurable reductions in stress-related physiological indicators? If physiological stress remains unchanged despite the absence of explanations, this would lend empirical support to the “black-box acceptability” hypothesis—suggesting users may tolerate opacity without sustained cognitive or physiological strain.

To address these questions, this study employs a within-subjects, single-factor experimental design with three levels of visualization transparency. The independent variable, visualization level, comprises: Level 0 (Black-box): No system state information provided; Level 1 (Standard): Basic system status feedback; Level 2 (Enhanced): Rich, explainable visualizations of system intent and reasoning. During simulated autonomous driving tasks, we collected multimodal data including eye-tracking metrics, behavioral interventions, and validated scales measuring technology acceptance and trust. This design aims to answer two core research questions: (1) Compared to a black-box system, does explainable visualization significantly enhance users’ self-reported trust? (2) In the absence of explanations, can users maintain low physiological stress levels—suggesting passive acceptance rather than active distrust?

The main contributions of this study are as follows:

- Through a high-fidelity simulated driving experiment, we systematically compare the effects of multiple levels of system transparency on users’ self-reported perceptions and behavioral responses;
- We employ a multimodal measurement framework—including visual attention patterns (eye-tracking), intervention behaviors, and subjective trust scales—to uncover how different levels of visualization influence trust formation in autonomous driving contexts;
- By integrating quantitative experimental data with qualitative insights from post-task interviews, we identify the distinct strengths and limitations of each visualization level, offering actionable guidance for the design of human-centered explainable interfaces in autonomous systems.

## 2. Materials and Methods

### 2.1. Participants

To ensure statistical reliability, an adequate sample size of qualified participants was determined a priori using the GPower 3.1.9.7 software. Power analysis indicated that with an effect size of *f* = 0.25, α = 0.05, and a target power of 0.80, a minimum sample size of 28 participants was required. The achieved power with *N* = 28 was 0.812.

To account for potential data attrition and further safeguard result robustness, we recruited 30 participants. During data preprocessing, eye-tracking data quality was evaluated, and one participant was excluded due to poor signal accuracy. The final analytical sample thus comprised 29 participants (16 female, 13 male), reflecting intentional recruitment to ensure gender diversity. Participants ranged in age from 18 to 60 years (*M* = 26.31, *SD* = 7.42).

Eligibility criteria required all participants to: Hold a valid driver’s license; Have normal or corrected-to-normal visual acuity; Possess normal hearing; Exhibit no color blindness or color vision deficiency.

All procedures were reviewed and approved by the Institutional Review Board of Nanjing University of Science and Technology. Written informed consent was obtained from each participant prior to the experiment. Upon completion, participants received a compensation of CNY 15.

### 2.2. Experiment Environment

This study employs a high-fidelity autonomous driving simulation system built upon the open-source CARLA platform (0.9.13 software), utilizing the Town10HD high-definition urban map as the foundational virtual environment. Dynamic scenario generation is implemented via CARLA’s Python API, enabling parametric control over environmental and behavioral variables. Experimental tasks are categorized into two conditions: Basic Task: Path-following under normal traffic flow; Complex Task: Reactive obstacle avoidance triggered by sudden events.

Scenario parameters—including obstacle placement and weather conditions—are dynamically configured using probabilistic distribution-based randomization algorithms to ensure ecological validity and inter-trial variability.

Leveraging the Unreal Engine 4.2 rendering backend, the system synchronously simulates dynamic environmental elements (e.g., time-of-day lighting, precipitation) and static infrastructure (e.g., road network topology). This provides a standardized, reproducible experimental environment for studying human trust in autonomous driving systems.

Each simulated driving trial begins with the vehicle starting from a non-motorized lane and autonomously navigating from Point A to Point B along a pre-defined high-precision GPS route. The ego-vehicle strictly adheres to virtual traffic regulations while dynamically encountering pedestrians, bicycles, and unexpected obstacles to evaluate system decision-making under complex urban conditions. Task completion is marked by successful autonomous parking at a designated residential zone. Prior to the experiment, participants were instructed to familiarize themselves with the simulated environment by manually driving the vehicle freely within the map for 10 min. Each trial spans approximately 2 km in distance and is constrained to under 3 min in duration. The total duration of the three experimental trials, including eye tracker calibration, was approximately 15 min. This duration was determined based on a pilot study (*N* = 7), which confirmed that it was sufficient to elicit meaningful trust assessments and intervention behaviors while minimizing eye fatigue (mean subjective fatigue rating <2 on a 5-point scale).

Participants interacted with the simulation via an immersive virtual driving setup. A Logitech G923 force-feedback steering wheel was employed to realistically replicate the tactile and kinesthetic feedback of steering during autonomous operation. Visual stimuli were presented across a multi-display setup: Two 4K UHD (3840 × 2160, 60 Hz, U270Q, ZLAU Corp., Guangzhou, China) monitors arranged to provide a 120° horizontal field of view, closely approximating natural human peripheral vision during driving; One 2K touchscreen simulating the vehicle’s center console interface, enabling real-time status monitoring and interactive control (e.g., mode switching, visualization toggling).

**Figure 1:**
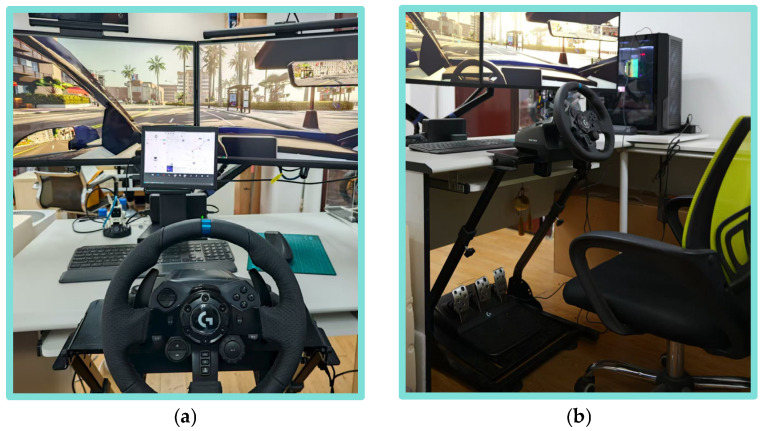
Experimental environment and approach: (**a**) view; (**b**) equipment.

Visual attention was recorded using the Tobii Pro Glasses 3 mobile (Tobii AB, Stockholm, Sweden) eye-tracking device. Equipped with a binocular infrared tracking system (50 Hz sampling rate, spatial accuracy <0.5°), the device continuously captured gaze coordinates, saccadic trajectories, and pupil diameter dynamics. Participants viewed the simulated environment through the device’s integrated head-mounted display (FOV: 46° × 34°), enabling precise spatiotemporal alignment between gaze behavior and environmental context (see Figure 1a).

Manual intervention behaviors—specifically braking actions—were captured via a Logitech G920 pedal (Logitech, Lausanne, Switzerland) unit with nonlinear response characteristics (see Figure 1b). Integrated with a high-resolution displacement sensor (0.1 mm resolution) and haptic feedback module, the pedal quantified brake depression depth (0–100 mm) in real time. Data were streamed via USB 2.0 to the host computer, where a custom Python 3.10.11 software script synchronized pedal inputs with the CARLA simulation engine, mapping user interventions to longitudinal vehicle control commands. This architecture enabled fine-grained monitoring of the dynamic interplay between human override and autonomous system behavior.

In CARLA, synchronous mode was enabled to ensure that all sensor data were strictly aligned to the same simulation timestamp. Additionally, data streams from CARLA, the Logitech G923 throttle/brake pedals, and the eye tracker were all timestamped using a monotonic system clock. A software-based synchronization marker was inserted at the beginning of each trial, enabling post hoc alignment of eye-tracking, vehicle control, and simulation events with sub-50-millisecond precision.

### 2.3. Scenario

**Figure 2:**
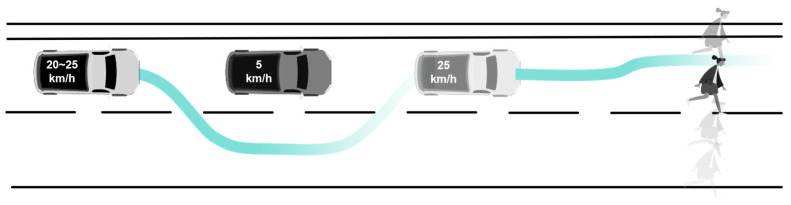
Schematic diagram of task process.

This experiment is based on typical urban driving scenarios from the i-VISTA Intelligent and Connected Vehicle Challenge, implemented within the Town10HD high-definition urban map of the CARLA platform, constructing an autonomous driving task from Point A to Point B. The experimental route spans 1 km and passes through 3 traffic lights and 6 intersections. During autonomous driving, the vehicle performs the following maneuvers: starting from standstill, merging into the main road, proceeding through an intersection under a flashing yellow light, decelerating to yield at pedestrian crosswalks, executing consecutive lane changes, exiting the main road, navigating narrow road segments, and performing curb-side parking (see Figure 2).

In addition, two non-routine events are incorporated. Based on typical accident patterns associated with automated driving assistance functions, the following two non-routine events are included to simulate unexpected disturbances in real-world driving: an overtaking event and a pedestrian avoidance event.

The overtaking event is triggered when the lead vehicle suddenly decelerates (speed drops to 5 km/h), testing the system’s real-time perception of dynamic obstacles and its path planning capability. The technical requirement specifies that, on a road with a 20 km/h speed limit, the test vehicle must overtake at 25 km/h, with lateral acceleration satisfying a <0.13 m/s^2^ to ensure ride comfort [40].

The pedestrian avoidance event is triggered when a pedestrian suddenly crosses from the roadside into the crosswalk (at a speed of 1.2 m/s), evaluating the system’s ability to detect vulnerable road users in unstructured scenarios. The technical requirement specifies that the braking response time must be <1.5 s, and the minimum safety distance must exceed 1.5 m [41].

### 2.4. Measurement

To systematically measure and compare the effects of different multi-level visualization methods on user experience—including both self-reported perceptions and behavioral responses—this study designed a Likert-scale questionnaire grounded in the Technology Acceptance Model (TAM). The questionnaire captures users’ self-reported trust perceptions toward the visualization system, as well as their subjective evaluations of its functional effectiveness (perceived usefulness) and operational convenience (perceived ease of use).

In parallel, users’ actual intervention behaviors during task execution were logged, and eye-tracking metrics were collected to objectively quantify cognitive processes and interaction patterns. By integrating self-reported questionnaire data with objective behavioral measures, this study enables a comprehensive assessment of how different visualization designs influence trust formation and user interaction behavior.

#### 2.4.1. Technology Acceptance Questionnaire Survey

TAM, introduced by Davis [42], serves as a foundational theoretical framework for understanding user adoption of information technology. According to TAM, an individual’s behavioral intention to use a technology is primarily shaped by Perceived Usefulness (PU) and Perceived Ease of Use (PEOU), with PEOU also positively influencing PU. This behavioral intention, in turn, directly predicts actual system usage.

In high-risk, highly interactive intelligent systems such as autonomous driving, perceived usefulness and trust are widely recognized as key determinants of users’ willingness to adopt autonomous vehicles [38]. The degree of trust users place in the system directly shapes their assessment of its functional effectiveness (i.e., PU) and their perception of operational complexity (i.e., PEOU). Higher levels of trust enhance users’ confidence in the system’s capabilities, thereby increasing perceived usefulness and alleviating concerns about usability. Conversely, diminished trust substantially undermines adoption intention. Thus, trust not only acts as an antecedent within the TAM framework but also indirectly influences behavioral intention via PU and PEOU, functioning as a crucial moderating or mediating variable in modeling user acceptance of autonomous driving technology [43]. Incorporating trust into the TAM framework offers a robust theoretical foundation for evaluating user experience and adoption intention in autonomous driving systems.

Guided by the Technology Acceptance Model, this study developed questionnaire items to assess users’ perceptions. Specifically, Q1 (“This system helps me complete driving tasks”) and Q2 (“The functions provided by this system are useful”) were designed to measure Perceived Usefulness (PU)—reflecting users’ subjective assessment of the technology’s ability to enhance task performance. Q3 (“I find this system difficult to use”) and Q4 (“I need to invest considerable effort to understand the system’s prompts”) were formulated to evaluate Perceived Ease of Use (PEOU)—capturing users’ perceived cognitive effort in operating the system. Notably, Q3 is reverse-scored. All items utilize a five-point Likert scale to systematically quantify subjective attitudes, facilitating a structured analysis of user acceptance toward multi-level visual driving assistance systems. To verify semantic appropriateness, we invited human factors (*N* = 7), engineering experts to conduct a content validity index (CVI) assessment, yielding a CVI score of 0.88. Additionally, a pre-test was carried out, which demonstrated a Cronbach’s α coefficient of 0.82.

This study integrates subjective and objective data through Spearman’s rank-order correlation analysis: each item score from the TAM questionnaire (Q1–Q4) is pairwise correlated with eye-tracking metrics (fixation entropy, mean pupil diameter) and behavioral measures (intervention frequency, reaction time) to assess the consistency between users’ self-reported perceptions and their implicit cognitive–behavioral responses.

#### 2.4.2. Intervention Behavior Metrics

This study developed a data acquisition module using Python to capture operational input signals from the autonomous driving simulation system in real time, with a focus on recording activation timestamps and pedal depression sequences of the brake pedal in the driving simulator. By analyzing the temporal sequence of these signals, intervention behaviors are identified through cycle detection: a complete “press–hold–release” process is defined as one intervention cycle. An effective intervention cycle is recognized when the system detects the pedal signal rising from zero (or near-zero) to exceed a preset activation threshold, and subsequently returning to a near-zero level. Based on this, the following key behavioral metrics are calculated: (1) Number of Intervention Instances —the total number of times users actively take over control during the experimental period; (2) Intervention Duration —the time span from the start to the end of each intervention, used to evaluate the persistence of interventions; (3) Average Intervention Intensity—quantifies the severity of user operations by computing the time-weighted average brake intensity during each intervention. The calculation formulas are as follows:

(1) $$\bar{B}=\frac{\sum_{i=1}^{n}B_{i}\cdot \Delta t_{i}}{\sum_{i=1}^{n}\Delta t_{i}}$$

In Equation (1), $\bar{B}$ denotes the time-weighted average brake intensity across all intervention events; $B_{i}$ is the instantaneous or mean brake intensity observed during the *i*-th intervention cycle; $\Delta t_{i}$ represents the temporal duration of that cycle; and *n* is the total count of distinct intervention intervals recorded during the experiment.

#### 2.4.3. Visual Behavior Metrics

In this study, we designed three visualization conditions with varying levels of system transparency to systematically investigate how information presentation influences user trust and cognitive load. Level 0 (Black-box) displayed only the basic road driving scene, providing no information about the autonomous system’s status or intent. Level 1 (Standard) presented conventional driving information—such as vehicle speed and navigation cues—on the center console display located to the right of the steering wheel. Level 2 (Enhanced) extended the standard condition by overlaying an augmented reality (AR) semi-transparent blue dashed trajectory (extending 10 m ahead) onto the windshield and adding multimodal sensor visualizations—including LiDAR point clouds, 360° camera views, and semantic segmentation outputs—on the right-side console (see Table 1). All interface elements were positioned and sized to ensure readability and comply with the human–computer interaction efficiency principles described by Fitts’ Law.

**Figure 3:**
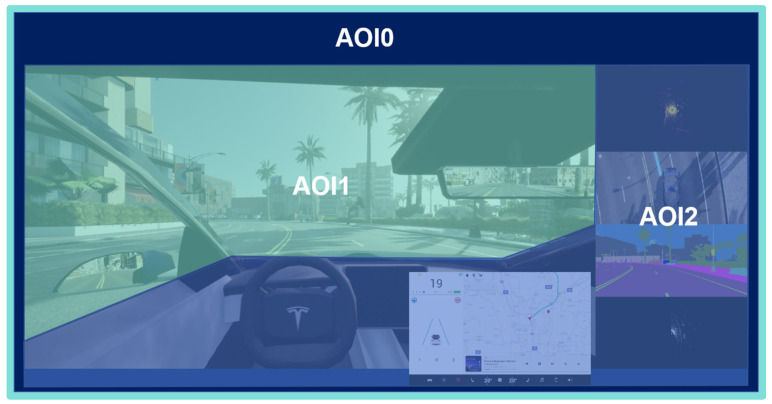
AOI definition.

Considering the impact of visualization on the interactive characteristics of the entire autonomous driving system, the eye-tracking Area of Interest (AOI) was divided into three primary regions, as illustrated in Figure 3: AOI0 encompasses the entire cockpit view, including both the external driving scene and the interior cabin; AOI1 focuses on the external view, covering the roadway and side/rearview mirrors; and AOI2 focuses on the interior cabin, specifically the steering wheel and the in-vehicle visual interface (see Figure 3).

Drawing on prior studies on eye-tracking in autonomous driving, we selected six visual behavior metrics: Number of Fixations, Fixation Duration (or Average Fixation Duration), Number of Glances, Number of Saccades within AOI, and Average Pupil Diameter. These metrics are detailed in the Table 2 below.

#### 2.4.4. Procedure

Each participant was required to complete one experimental trial with each autonomous driving system, resulting in a total of three driving sessions per participant. To control for potential learning effects, the order in which participants used the different systems was randomized across individuals. Each experimental trial lasted approximately 3 min, and the entire experimental procedure—including system interaction, questionnaire completion, and post-task interviews—took about 20 min in total, thereby minimizing the risk of fatigue effects [49]. The experimental procedure was conducted as follows.

Information Collection: Upon arrival at the laboratory, participants were provided with an informed consent form. They then completed a demographic questionnaire, reporting their age range, gender, and years of driving experience. Additionally, they filled out a pre-experiment questionnaire to assess their initial level of trust in autonomous driving systems.

Training: Participants received a brief training session to ensure familiarity with the simulated driving platform, including manual vehicle control and the operational tasks required during the experiment. A short driving test was administered to confirm that participants could successfully navigate to a destination using manual control.

Eye-Tracker Calibration: Prior to the experimental trials, participants were assisted in calibrating the eye-tracking system using a standard calibration card.

Experimental Trials: Each participant completed three driving trials. During each trial, they encountered two unexpected (non-routine) events. The order of the autonomous driving systems and the timing of the non-routine events were randomized across trials to mitigate memory or order effects. Participants were not informed in advance about the number or frequency of these events.

Post-Experiment Interview: After completing all three trials, participants completed TAM-based questionnaire. This was followed by a semi-structured interview to collect qualitative feedback and self-reported evaluations regarding their experience with the systems.

## 3. Results

All data were first subjected to validity screening. The internal consistency of the questionnaire was assessed using Cronbach’s alpha, and visual behavior data were retained for analysis only when eye-tracking accuracy exceeded 90%. One participant (3.3%) was excluded from the analysis due to having less than 85% valid data. No imputation was performed for missing values; instead, the listwise deletion method was employed. Subsequently, statistical analyses were conducted using IBM SPSS Statistics (Version 26.0; IBM Corp., Armonk, NY, USA) to examine the relationships between the independent variable (three levels of visualization transparency) and the dependent variables (questionnaire responses, behavioral measures, and eye-tracking metrics).

The Shapiro–Wilk test was used to assess the normality of the dependent variables. Given that each participant experienced all three transparency conditions, a one-way repeated-measures analysis of variance (ANOVA)was performed when the normality assumption was satisfied, followed by Bonferroni-corrected pairwise comparisons. For Mauchly’s test of sphericity, the significance value under the assumption of sphericity was used when the assumption was met. If the sphericity assumption was violated, the Greenhouse–Geisser correction was applied to adjust the degrees of freedom and associated *p* values.

When the normality assumption was not met, the non-parametric Friedman test was used as an alternative. In cases where the Friedman test indicated significant differences, post hoc pairwise comparisons were conducted using the Wilcoxon signed-rank test with Bonferroni correction for multiple comparisons. All statistical tests were evaluated at a significance level of *p* < 0.05.

Some figures in this paper were generated using ChiPlot (https://www.chiplot.online/) (accessed on 10 August 2025), including the correlation heatmap, violin plot, and box plot.

### 3.1. Self-Reported Evaluation

The questionnaire data did not meet the assumption of normality; therefore, the Friedman test was employed to assess whether there were significant differences in participants’ self-reported ratings of perceived usefulness and perceived ease of use across the three levels of information presentation (Level 0, Level 1, and Level 2). The results are summarized in Table 3.

As shown in Table 3, significant differences were observed for Q1 (χ^2^ = 35.489, *p* < 0.01), Q2 (χ^2^ = 25.371, *p* < 0.01), and Q3 (χ^2^ = 10.299, *p* < 0.01). In contrast, Q4 did not reach statistical significance (χ^2^ = 2.605, *p* > 0.05), indicating that participants’ responses to the statement “I need to expend considerable mental effort to understand the system’s prompts” did not differ significantly across the three information presentation levels.

**Figure 4:**
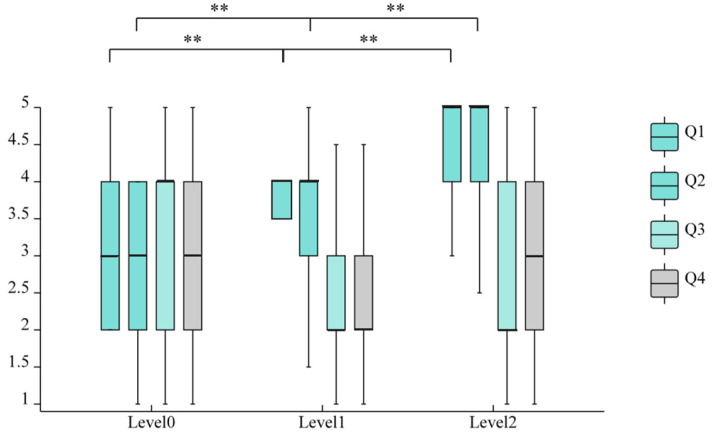
Boxplot of Self-Reported Technology Acceptance Evaluation Scale for Three-Level Visualization (** *p* < 0.01).

To explore significant omnibus effects, post hoc pairwise comparisons were conducted using the Wilcoxon signed-rank test (see Figure 4), with *p*-values adjusted via the Holm–Bonferroni procedure (family-wise α = 0.05; number of comparisons = 6:3 questions × 2 pairwise contrasts each). For perceived usefulness (Q1 and Q2), significant differences were observed between Level 1 and Level 0 (adjusted *p*-values, *p_adj_* < 0.001) and between Level 1 and Level 2 (*p_adj_* < 0.001), indicating that users’ evaluations of functional effectiveness varied significantly across information load levels. Specifically, increased visual information density correlated with higher perceived system effectiveness in supporting driving tasks (*M*_Level 1_ = 4.3 vs. *M*_Level 0_ = 3.1; *M*_Level 2_ = 3.6), reflecting enhanced functional utility.

In contrast, perceived ease of use Q3 showed no significant differences between Level 1 and Level 0 (*p* = 0.288) or between Level 1 and Level 2 (*p* = 0.172). Q4 did not reach statistical significance (χ^2^ = 2.605, *p* > 0.05), suggesting that information density had no significant effect on perceived mental effort. These differences remained non-significant before and after correction (*p_adj_* = 0.34). Although the median score for Q3 was numerically higher under Level 2 (median = 3.0) than Level 1 (median = 2.0) on the 5-point Likert scale (with higher values indicating greater difficulty), this difference did not reach statistical significance. Therefore, we cannot conclude that increased information density meaningfully affected perceived ease of use.

However, a significant increase in cognitive effort was observed for Q4 when comparing Level 2 to Level 1 (*p* < 0.001), indicating that participants found the system prompts more mentally demanding under enhanced visualization. This suggests a potential trade-off: while functional usefulness improved (as shown in Q1–Q2), the added information may have imposed a higher cognitive cost, even if overall ease-of-use ratings did not change significantly.

### 3.2. Intervention Behavior

**Figure 5:**
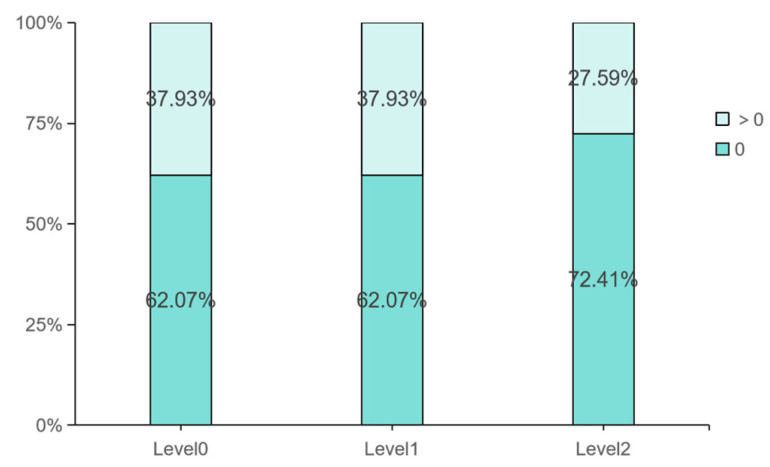
Distribution of Participants’ Intervention Behaviors.

Over 50% of participants made no interventions across all tasks, yielding a right-skewed distribution of intervention frequency (see Figure 5). While approximately 38% of participants intervened at least once under Level 0 and Level 1, this proportion dropped significantly to 27.59% under Level 2. Given the non-normal distribution, nonparametric tests were used: a Friedman test to assess overall differences in intervention frequency across the three visualization levels, followed by Wilcoxon signed-rank post hoc pairwise comparisons.

The Friedman test results revealed that no statistically significant differences were observed in intervention frequency (χ^2^ = 2.085, *p* > 0.05), intervention duration (χ^2^ = 0.792, *p* > 0.05), or average intervention intensity (χ^2^ = 2.528, *p* > 0.05) across the three information presentation levels, as shown in Table 4.

Further analysis using the Wilcoxon signed-rank test for paired samples was conducted to pairwise comparisons between visualization levels on intervention frequency, intervention duration, and average intervention intensity. As shown in Table 5, no statistically significant differences were observed between Level 1–Level 0 across all three metrics (*p* = 0.856, *p* = 0.397, *p* = 0.510). For Level 1–Level 2 comparisons, non-significant differences were found in intervention frequency (*p* = 0.101) and intervention duration (*p* = 0.084). Notably, the uncorrected *p*-value for average intervention intensity (Level 1 vs. Level 2) was 0.031, but this did not survive multiple comparison correction (*p_adj_* = 0.186) and should not be interpreted as evidence of a true effect, especially in the absence of a significant global test.

### 3.3. Visual Behavior

**Figure 6:**
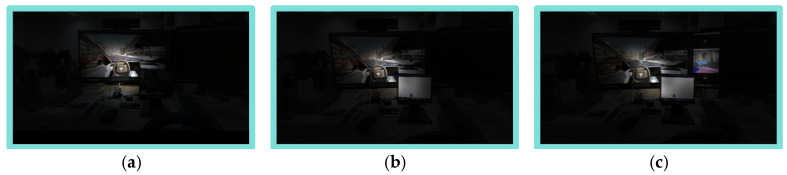
Visual behavior of drivers under different levels of information during autonomous driving tasks: (**a**) Level 0; (**b**) Level 1; (**c**) Level 2.

We employed eye-tracking heatmaps to visualize drivers’ attention distribution across different levels of information visualization systems. The heatmaps utilized transparency gradients to indicate visual focus intensity (high transparency reflecting high attention, low transparency/black representing low attention). As illustrated in Figure 6, participants’ attention was predominantly concentrated on road areas within the driving simulation environment. With increased visualization levels, attention distribution transitions from focused to dispersed patterns.

Shapiro–Wilk normality tests were conducted on the collected visual behavior data. As shown in Table 6, significant deviations from normality (*p* < 0.05) were observed for total fixation duration, average fixation duration, average pupil diameter, and saccade frequency, necessitating nonparametric analyses for these metrics. However, saccade frequency and fixation count within AOIs across Level 0, Level 1, and Level 2 conditions did not show significant deviations (*p* > 0.05), indicating normal distribution and satisfying parametric test assumptions.

**Figure 7:**
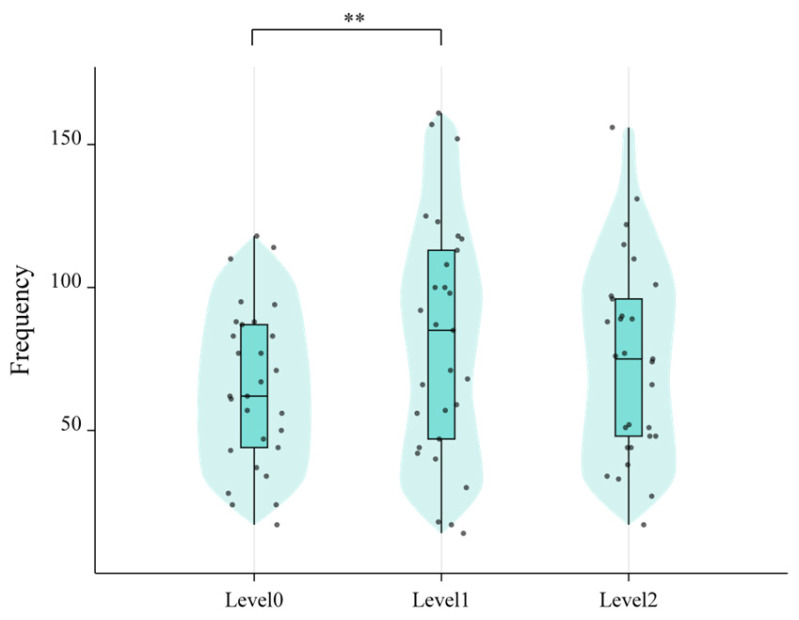
Paired Comparisons of Saccade Frequency in AOIs Across Three-Level Visualization Conditions (** p < 0.01).

Paired-samples t-tests were conducted to examine differences between visualization levels in saccade frequency and fixation count within AOIs (see Figure 7). Results revealed that saccade frequency in Level 0 was significantly lower than in Level 1 (*p* < 0.05), as illustrated in Figure 7. However, no significant differences were observed between Level 1 and Level 2 for either metric.

The Friedman test revealed statistically significant differences across information presentation levels for total fixation duration (χ^2^ = 16.828, *p* < 0.001), average fixation duration (χ^2^ = 7.103, *p* = 0.029), and average pupil diameter (χ^2^ = 38.345, *p* < 0.001), as presented in Table 6. No significant effect was observed for number of glances (χ^2^ = 0.538, *p* = 0.764); thus, it was excluded from post hoc analysis.

**Figure 8:**
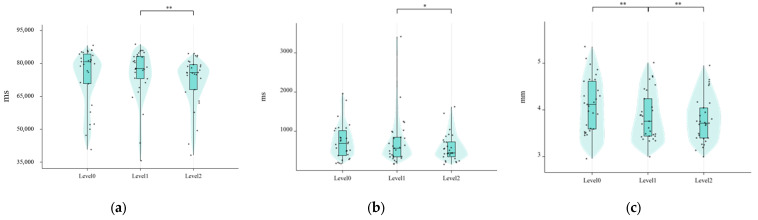
Paired Comparisons of Visual Behavior Across Three-Level Visualization Conditions: (**a**) Total duration of fixations; (**b**) Average duration of fixations; (**c**) Average pupil diameter (* *p* < 0.05; ** *p* < 0.01).

For the three significant metrics, post hoc pairwise comparisons were conducted using Wilcoxon signed-rank tests (see Figure 8), with *p*-values adjusted via the Holm–Bonferroni procedure (family-wise α = 0.05; 6 comparisons: Level 1 vs. Level 0 and Level 1 vs. Level 2 for each metric). Additionally, for the normally distributed metric saccade frequency within AOIs, paired *t*-tests with Holm–Bonferroni correction (2 comparisons) were performed. After correction, the following differences remained statistically significant (see Table 7)

Notably, the uncorrected difference in average fixation duration between Level 1 and Level 2 (*p* = 0.048) did not survive Holm–Bonferroni correction (*p_adj_* = 0.096) and is therefore not considered statistically reliable. Similarly, no significant differences were observed in any metric for the Level 1 vs. Level 0 contrast except for pupil diameter and saccade frequency.

Collectively, these results demonstrate that Level 1 uniquely modulates visual behavior: it elicits the highest pupil dilation (a proxy for cognitive effort), prolongs total visual engagement relative to Level 2, and increases saccadic activity relative to Level 0. In contrast, Level 2 does not confer additional visual processing benefits over Level 1, and may even reduce sustained attention (as reflected in shorter total fixation duration). The absence of significant effects for average fixation duration and number of glances further underscores the metric-specific nature of visualization impacts on eye movement behavior.

### 3.4. Correlations Between Subjective and Objective Measures

**Figure 9:**
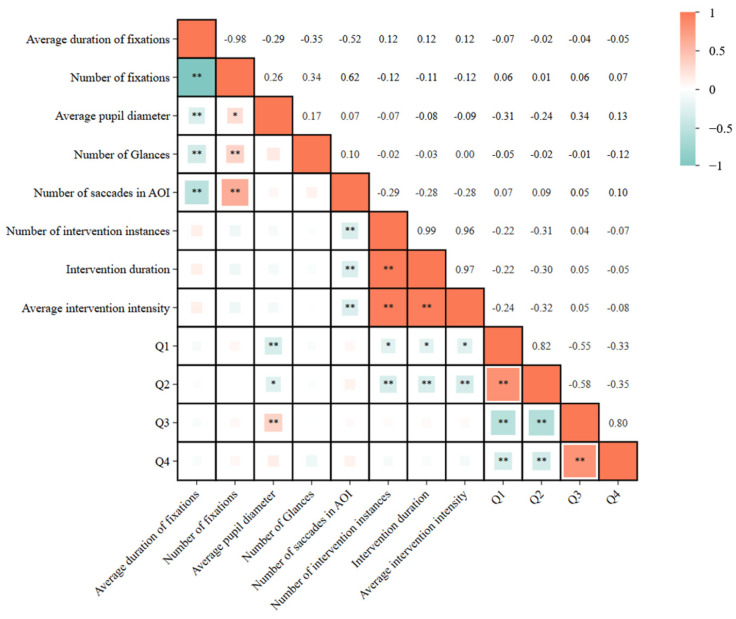
Spearman’s Correlation Matrix of Self-Reported Ratings and Physiological Response Metrics (* *p* < 0.05; ** *p* < 0.01).

We further examined the association between subjective trust ratings and objective behavioral measures. Spearman’s correlations revealed associations between self-reported ratings (Q1–Q4) and physiological response metrics, accommodating non-normal or ordinal data distributions, as shown in Figure 9. Results indicated significant negative correlations between Q1/Q2 and both average pupil diameter (a cognitive load indicator) and intervention behavior metrics. Conversely, Q3 demonstrated a significant positive correlation with average pupil diameter. These findings suggest that as perceived system usefulness increases, cognitive workload or physiological arousal decreases, accompanied by reduced intervention intentions. Notably, perceived ease of use exhibited a significant positive correlation with average pupil diameter, though the underlying mechanisms remain to be explored.

## 4. Discussion

In this experiment, by collecting participants’ eye-tracking data, behavioral responses, and results from the Technology Acceptance Questionnaire during the autonomous driving task, we obtained the following findings and systematically investigated two core research questions.

Self-reported Evaluation: Participants rated the system significantly more favorably under Level 2 than under Level 0 or Level 1 on Q1 (“The system helps me complete driving tasks”) and Q2 (“The system’s functions are highly practical”), with Holm–Bonferroni-corrected *p*-values < 0.01 for both comparisons. Q3 (“I find this system difficult to use”) showed the lowest (most positive) scores in Level 2, indicating improved perceived usability. In contrast, Q4 (“I need to expend significant effort to understand system notifications”) did not differ significantly across transparency levels (*p_adj_* > 0.05), although the median response was highest (least favorable) under Level 2.

Intervention behavior: Overall intervention frequency was low across all conditions. No statistically significant differences were observed in intervention frequency (Friedman χ^2^ = 2.528, *p* = 0.282) or total intervention duration across the three transparency levels. The comparison between Level 1 and Level 2 yielded an uncorrected *p*-value of 0.031 for average intervention intensity, but this effect did not remain significant after Holm–Bonferroni correction (*p_adj_* = 0.186).

Visual Behavior: Eye-tracking metrics revealed significant omnibus effects of transparency level on total fixation duration, average fixation duration, and average pupil diameter (all *p* < 0.05). Post hoc pairwise comparisons showed that total fixation duration was significantly lower in Level 2 than in Level 1 (*p_adj_* = 0.030), and average pupil diameter was also significantly lower in Level 2 compared to Level 1 (*p_adj_* = 0.018). Saccade frequency within regions of interest was significantly higher in Level 1 than in Level 0 (*p_adj_* = 0.006), with no significant difference between Level 1 and Level 2.

### 4.1. Information Visualization Enhances User Trust Compared to Black-Box Systems

The significantly higher Q1 and Q2 scores under Level 2 suggest that providing sensor data and decision-making pathways enhances users’ perception of the system’s practical utility and reliability. This aligns with Lee and See’s [50] notion that process transparency fosters capability-based trust. Similarly, the improved Q3 scores indicate that well-designed visualizations can enhance subjective usability.

However, the lack of significant improvement in Q4—coupled with a median increase in perceived effort under Level 2—suggests that explanatory richness may introduce complexity that offsets usability gains. This pattern resonates with the concept of extraneous cognitive load [51,52] and Körber et al.’s [53] warning about “explanation fatigue,” where excessive detail may overwhelm rather than inform.

At the behavioral level, the absence of significant differences in intervention metrics—even after correcting for multiple comparisons—indicates that increased transparency did not translate into measurable changes in takeover behavior in this experimental context. This underscores a potential dissociation between subjective trust and actual behavioral reliance.

Eye-tracking results reveal a more nuanced picture. The reduced pupil diameter and total fixation duration under Level 2, despite higher information density, suggest a decrease in objective cognitive load. This contrasts with the subjective perception of increased complexity (Q4), pointing to a subjective–objective dissociation in cognitive load assessment. This pattern aligns with Hergeth et al. [54], who observed that higher trust in automation is associated with reduced visual monitoring during non-critical phases, reflecting greater reliance and lower vigilance demands. Concurrently, the efficiency of information processing under Level 2 may support the formation of a more accurate and stable mental model of system behavior, as suggested by Bauer et al. [55]. One possible explanation is that predictive visualizations (e.g., AR trajectories) enable more efficient information integration, allowing users to extract greater meaning with fewer cognitive resources—a principle consistent with cognitive load theory [56].

The elevated saccade frequency in Level 1 (vs. Level 0) implies that minimal transparency prompts active visual scanning, possibly due to ambiguity in system cues. In contrast, Level 2’s richer explanations may support information-per-saccade efficiency, reducing the need for compensatory scanning. Together, these findings suggest a nonlinear relationship between transparency level and cognitive demand: moderate-to-high transparency, when well-structured, can reduce load despite greater information volume.

These results collectively highlight that trust, usability, and cognitive load are multidimensional constructs that may diverge across measurement modalities. Future work should investigate individual difference factors (e.g., expertise, cognitive style) and dynamic task contexts that modulate these effects.

### 4.2. The Low Physiological Stress Caused by the Lack of Explanations Does Not Fully Support Trust

The findings demonstrate that the lack of system explanations in the black-box condition (Level 0) did not reduce physiological arousal—as indicated by elevated average pupil diameter (Level 0 > Levels 1/2, *p* < 0.05). While behavioral and eye-tracking metrics (e.g., saccade frequency, attention distribution) showed no statistically significant differences, their median values trended upward compared to Levels 1 and 2. Combined with self-reported data (lower Q1–Q3 scores), this suggests participants experienced heightened cognitive demand.

Although Level 0 showed a lower mean saccade frequency in AOIs compared to Level 1—an indicator often associated with reduced cognitive load—the integration of self-report evaluations and behavioral data suggests this low arousal state was not due to psychological relaxation, but rather reflected a passive, disengaged monitoring strategy [57]. Specifically, Q1–Q3 scores in Level 0 were significantly lower, indicating reduced user confidence in system functionality and operational convenience. Concurrently, eye-tracking data revealed the fewest saccades within regions of interest and the most restricted attention distribution under Level 0, reflecting minimal proactive exploration. This aligns with Endsley and Kiris’ [58] concept of “out-of-the-loop” states: due to the system’s lack of explainability, users failed to comprehend its behavioral logic, leading to reduced information seeking and cognitive investment, and ultimately adopting passive following strategies [59,60].

Crucially, the lowest pupil diameter was observed in Level 2—not Level 0—indicating that true cognitive ease arises from predictability and understanding, not from information absence. The slightly elevated pupil diameter in Level 1 (relative to both Level 0 and Level 2) further suggests that moderate but non-explanatory information may impose the highest cognitive demand, as users attempt to infer system intent without sufficient cues.

In summary, the absence of explanations does not maintain a healthy low-stress state. Instead, it risks undermining trust and triggering monitoring complacency, thereby weakening human–machine collaborative efficiency. A genuinely effective low-stress state should be built on predictability, controllability, and shared mental models—principles aligned with human-centered design [61,62]—which represent the core value provided by moderate, meaningful visual explanations.

## 5. Conclusions

This study reveals that the impact of information visualization on user experience in autonomous driving is nonlinear and context-dependent. Enhanced information visualization (Level 2) produces divergent effects across dimensions: it significantly improves perceived usefulness, user trust, and engagement in proactive monitoring—key enablers of safe and efficient human–machine collaboration. However, these benefits come at a cognitive cost, as Level 2 also leads to substantially higher self-reported cognitive load despite lower physiological indicators of cognitive load (e.g., decreased average pupil diameter). Critically, this added cognitive demand is not offset by improvements in perceived ease of use, suggesting that simply increasing information density does not yield uniformly positive outcomes.

In contrast, both the black-box condition (Level 0) and the standard visualization (Level 1) show limited differentiation across most measures, underscoring that not all increases in transparency are functionally meaningful. The findings therefore advocate for a shift from “more information” to “Meaningful Transparency”—a design paradigm that prioritizes contextually relevant, interpretable, and actionable explanations tailored to users’ situational needs and cognitive capacities, rather than maximal disclosure.

This study has several limitations. The participant sample was skewed toward younger female adults, which may limit generalizability. The simulated driving environment, while enabling controlled measurement, lacks real-world complexities such as unpredictable traffic dynamics, emergency events, or diverse interface layouts. Additionally, eye-tracking metrics like fixation count and saccade frequency are sensitive to variations in the size and placement of visual elements, potentially affecting their validity as cognitive state indicators. Future research should validate these findings in real-world or high-fidelity driving contexts, explore adaptive visualization strategies that modulate information presentation based on workload and task criticality, and examine how individual differences (e.g., age, expertise, cognitive style) influence responses to varying levels of system transparency. Although enhanced visualization significantly improved subjective trust and certain eye-tracking metrics, behavioral indicators did not show the expected reduction in intervention frequency or reaction time. Moreover, while Spearman’s rank-order correlations were computed between TAM item scores (Q1–Q4) and objective measures (fixation entropy, mean pupil diameter, intervention frequency, and reaction time), these analyses were based on the full set of valid participant observations (*N* = 29) and did not control for potential covariates such as age, driving experience, or prior exposure to automated systems. This limits our ability to isolate the unique contribution of visualization design from individual differences. A dissociation also emerged between self-reported assessments and eye-tracking metrics in the cognitive load dimension. Future research should explore behavioral threshold ranges, incorporate covariate-adjusted models (e.g., partial correlations or regression-based approaches), and further unravel the mechanisms underlying this subjective–objective dissociation.

## Figures and Tables

**Table 1:** Interface Visualization Conditions.

Level	Information Type	Picture
Level 0	No system transparency	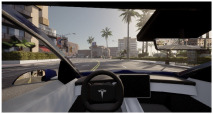
Level 1	Basic functional status (non-explanatory)	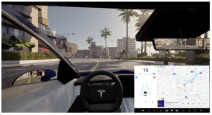
Level 2	Multimodal perception + behavioral intent (high transparency)	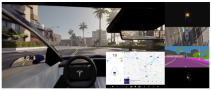

**Table 2:** Key eye movement indicators that reflect cognitive state and trust.

Metric	Trust-Related Pattern	Citation
Number of Fixations	When trust decreases, the number of fixations increases, and users monitor the system more frequently.	[44,45]
Fixation duration/average duration	When trust decreases, both the total fixation duration and the average fixation duration increase, indicating that users are exerting cognitive effort.	[44,45]
Number of Glances	When trust declines, the number of saccades increases, reflecting heightened user perception of anxiety.	[44]
Number of Saccades in AOI (Saccade frequency)	When trust decreases, the number of eye jumps within areas of interest increases, reflecting a cautious attitude of users who repeatedly cross-check information.	[46,47]
Average Pupil Diameter	An increase in average pupil diameter indicates heightened cognitive load in participants. This is often in conjunction with the number of saccades within the area of interest, reflecting a state of heightened vigilance in trust assessment.	[47,48]

**Table 3:** Friedman Test Results for the Technology Acceptance Questionnaire.

Metric	χ^2^	*p*
Q1	35.489	0.000 **
Q2	25.371	0.000 **
Q3	10.299	0.006 **
Q4	2.605	0.272

** *p* < 0.01.

**Table 4:** Friedman test results for intervention behavior across visualization levels.

Metric	χ^2^	*p*
Number of intervention instances	2.085	0.353
Intervention duration	0.792	0.673
Average intervention intensity	2.528	0.282

**Table 5:** Post hoc Wilcoxon signed-rank test results for pairwise comparisons of intervention metrics.

Group	Metric	Kruskal–Wallis (P25, P75)		ΔM	z	*p*
		Paired1	Paired2			
Level 1–Level 0	Number of intervention instances	0(0,1)	0(0,1)	0	0.182	0.856
	Intervention duration	0(0,1.6)	0(0,1.4)	0	0.847	0.397
	Average intervention intensity	0(0,0.3)	0(0,0.3)	0	0.659	0.510
Level 1–Level 2	Number of intervention instances	0(0,1)	0(0,1)	0	1.642	0.101
	Intervention duration	0(0,1.6)	0(0,0.8)	0	1.726	0.084
	Average intervention intensity	0(0,0.3)	0(0,0.1)	0	2.158	0.031 *

* *p* < 0.05

**Table 6:** Friedman Test Results for Visual Behavior Across Information Presentation Levels.

Metric	χ^2^	*p*
Total duration of fixations	16.828	0.000 **
Average duration of fixations	7.103	0.029 *
Average pupil diameter	38.345	0.000 **
Number of Glances	0.538	0.764

* *p* < 0.05; ** *p* < 0.01.

**Table 7:** Post hoc pairwise comparisons with Holm–Bonferroni-adjusted *p*-values.

Metric	Comparison	*p*_adj_
Total duration of fixations	L0 vs. L1 L1 vs. L2	0.120 0.030 *
Average duration of fixations	L0 vs. L1 L1 vs. L2	0.420 0.096
Average pupil diameter	L0 vs. L1 L1 vs. L2	0.000 ** 0.018 *

* *p* < 0.05; ** *p* < 0.01.

## Data Availability

In accordance with privacy and ethical considerations, as well as specific requests from a subset of participants, the data generated or analyzed in this study contain sensitive information and therefore cannot be made publicly available.

## Abbreviations

The following abbreviations are used in this manuscript:

XAI	eXplainable Artificial Intelligence
AI	Artificial Intelligence
AR	Augmented Reality
IBM Corp.	International Business Machines Corporation
USA	United States of America
NY	New York
AOI	Area of Interest
ANOVA	Analysis of Variance
TAM	Technology Acceptance Model
PU	Perceived Usefulness
PEOU	Perceived Ease of Use
